# Impact of Extreme Heat on Cardiovascular Health in Kuwait: Present and Future Projections

**DOI:** 10.1007/s44197-024-00330-5

**Published:** 2024-12-02

**Authors:** Yazan Alwadi, Ali Al-Hemoud, Haitham Khraishah, Fahd Al-Mulla, Petros Koutrakis, Hamad Ali, Barrak Alahmad

**Affiliations:** 1 Environmental Health Department, Harvard T.H. Chan School of Public Health, 401 Park Dr, 4th floor, Room 412G, Boston, MA 02115 USA; 2 Environment and Life Sciences Research Center, Kuwait Institute for Scientific Research, Kuwait City, Kuwait; 3 Division of Cardiovascular Medicine, Case Western Reserve University, Cleveland, OH USA; 4 Translational Research Department, Dasman Diabetes Institute, Kuwait City, Kuwait; 5 Department of Medical Laboratory Sciences, Faculty of Allied Health Sciences, Health Sciences Center, Kuwait University, Kuwait City, Kuwait; 6 Division of Nephrology and Hypertension, Mayo Clinic, MN, Rochester, USA

**Keywords:** Cardiovascular disease, Hospitalizations, Middle east, Climate change, Ischemic heart disease, Climate projections

## Abstract

**Background:**

The Middle East, especially Kuwait, is experiencing rapidly rising temperatures due to climate change. Cardiovascular diseases (CVD) are the leading cause of mortality in the country, and extreme heat is expected to exacerbate hospitalizations for cardiovascular diseases. There is limited data quantifying the historical and future impacts of heat on hospitalizations for cardiovascular diseases in Kuwait.

**Methods:**

We collected daily hospital admission data of cardiovascular diseases in Kuwait from 2010 to 2019. We modeled the relationship between temperature and cardiovascular disease hospitalizations using distributed lag non-linear models (DLNMs), adjusting for relative humidity and seasonality. Future temperature projections for Kuwait under moderate and extreme climate change scenarios were obtained from the Coupled Model Inter-comparison Project Phase 6 (CMIP6), and the impact on cardiovascular disease hospitalizations was extrapolated for every decade until 2099.

**Results:**

During the baseline period (2010–2019), a total of 263,182 CVD cases were recorded. Of which, 20,569 (95% eCI: 3,128, 35,757) were attributed to heat. We found that the relative risk of hospitalization for CVD increased from 1.292 (95% CI: 1.051, 1.589) at 41 °C to 1.326 (95% CI: 1.006, 1.747) at 43 °C, compared to the minimum morbidity temperature. Projections showed that, under moderate climate scenarios, CVD hospitalizations would increase by 1.96% by 2090–2099, while under extreme scenarios, the increase could reach 4.44%.

**Conclusions:**

Extreme heat significantly contributes to CVD hospitalizations in Kuwait. This burden is projected to increase under climate change. Findings highlight the urgent need for healthcare system preparedness to mitigate the future health impacts of rising temperatures in Kuwait.

## Introduction

The Middle East, a region covering approximately 7 million km² and home to over 490 million people [1], is rapidly heating and experiencing severe impacts from climate change [2]. Characterized by its arid and hyper-arid environments, this region faces unique challenges that threaten its ecological sustainability, human health and survivability [3–5].

Temperature projections for the region predict continuing upward trends and indicate that the frequency, intensity, and duration of extreme climatic events and heatwaves will continue to rise, posing even higher risk in the future [6–10]. A study examining the potential impacts of climate change in the region, based on future climate projection scenarios, estimated that the daily heat-attributable mortality could increase by 8 to 20 times by the year 2100 [11].

Kuwait, a Middle Eastern country known for being one of the hottest in the world, is facing the severe consequences of climate change, with temperatures reaching unprecedented highs. In the summer of 2016, the country recorded a scorching 54.0 °C [12], and it continues to experience frequent days with temperatures surpassing 40 °C and even 50 °C [13].

Studies have demonstrated that these extreme temperatures have had severe detrimental effects on human health and mortality in Kuwait [5, 14–17]. More specifically, that the overall mortality risk doubles at 41 °C and triples at 42 °C [18]. These findings highlight the immediate health challenges posed by the extreme climate conditions prevalent and projected in the country.

Cardiovascular diseases are the leading cause of mortality worldwide. In Kuwait, cardiovascular diseases are also the leading cause of mortality with almost 58 and 12 deaths for every 100,000 population are attributed to ischemic heart disease (IHD) and stroke, respectively [19]. Diabetes is thought to be the leading risk factor for developing cardiovascular disease in Kuwait [20]. One national self-reporting survey in Kuwait estimated that approximately one-in-ten of surveyed subjects were at elevated 10-year risk of developing both type 2 diabetes and chronic heart disease [21]. A healthcare-based survey in the country estimated that individuals with heart disease are linked to a 5-fold increase in hospital utilization rates compared to those who do not have heart disease [22].

The relationship between climatic conditions and cardiovascular diseases is well established in the literature [23]. With predictions indicating a significant rise in temperatures due to climate change, there is a pressing need to understand how climate change could exacerbate heart disease rates, particularly hospitalizations and not just mortality. Currently, there is a lack of local data estimating the impact of increasing temperatures on the incidence of heat-related hospitalizations for heart diseases.

In this study we: (1) establish a baseline time series of cardiovascular disease hospitalizations due to extreme heat in Kuwait, and (2) estimate the potential impact of climate change on cardiovascular disease hospitalizations for every decade until 2099 in Kuwait.

## Methods

Daily data on hospitalizations for cardiovascular diseases were collected from the National Centre for Health Information, Department of Vital Statistics, Ministry of Health, Kuwait, for the period from January 1, 2010, to December 31, 2021. This dataset includes all hospital admissions for cardiovascular diseases occurring within the country, categorized according to the International Classification of Diseases, 10th version (ICD-10) codes I00-I99, with further stratification by ischemic heart disease (IHD; I20-I25) and stroke (I60-I69). COVID years (2020 and 2021) were excluded from the analysis due to major disruptions to the healthcare system.

Daily ambient temperature and relative humidity measurements were obtained from the meteorological services at Kuwait Airport. These measurements represent 24-hour averages of temperature (in degrees Celsius) and relative humidity (in %) for the same study period.

Simulations of daily average temperatures in Kuwait through 2099 were sourced from the Copernicus Climate Change Service, and output from the Coupled Model Inter-comparison Project Phase 6 (CMIP6), from the IPCC’s 6th assessment report [24, 25]. We selected two climate change scenarios for analysis: a moderate scenario with a projected radiative forcing of 4.5 watts per square meter (W/m^2^) by 2100 (SSP2-4.5) and an extreme scenario with 8.5 W/m^2^ by the same year (SSP5-8.5). From the CMIP6, 18 models were chosen based on their ability to provide historical and future temperature simulations for Kuwait under these scenarios. These projection models were described in more details elsewhere [15, 25]. These temperature projections were downscaled and bias-corrected for Kuwait using monthly averages from observed data to maintain accuracy in trends and variability [26]. A baseline decade of 2000–2009 was used to assess potential climate impacts [15].

From 2010 to 2019, we examined the relationship between temperature and cardiovascular disease hospitalizations in Kuwait using distributed lag non-linear models (DLNMs), a flexible modeling framework ideal for estimating non-linear and delayed associations [27]. At baseline, we fitted quasi-Poisson regression, adjusting for relative humidity, seasonality, time trends, and day of the week. Similar to previous DLNM models [27, 28], temperature was modelled with natural splines and 3 degrees of freedom placed at the 10th, 75th and 90th percentile. The lag was modelled with natural splines and 3 degrees of freedom placed equally at the log scale for 30 days, following a previous temperature paper in Kuwait [14]. Historical relative risks of hospitalization for each cause were reported for extreme hot temperatures (41, 42, 43 °C) compared to a minimum morbidity temperature (MMT). Relative humidity was modelled linearly and without a lag [29]. Our projections applied log-linear extrapolation (of the natural spline) of the baseline relationship into future decades by extending the timeseries of observed exposure-response into potential future temperatures under climate change scenarios [30]. The analysis did not assume any demographic changes or adaptation measures. This approach helped isolate the effects of climate from other trends in future cardiovascular disease hospitalizations in Kuwait. We reported attributable risk of extreme heat at baseline and in future decades. The temperature-related attributable number of hospitalizations is calculated as follows [30]:

$$\eqalign{ Hos{p_{attr}}\, & = Hos{p_{tot}} \cr & \times \>(1 - {e^{ - \left( {f\left( {{T_{mod}};\>\theta {\>_b}} \right) - s\left( {{T_{mm}};\>\theta {\>_b}} \right)} \right)}}) \cr} $$

Where $\:{Hosp}_{attr}$ is the number of hospitalizations attributable to hot temperatures, and $\:{Hosp}_{tot}$ represents the total number of hospitalizations. The functions $\:f\left({T}_{mod};\:{\theta\:}_{b}\right)$ and $\:s\left({T}_{mm};\:{\theta\:}_{b}\right)$ represent the exposure-response functions for the modified projected temperature series $\:{T}_{mod}$ and the minimum morbidity temperature threshold $\:{T}_{mm}$. Empirical confidence intervals (eCI) around the effect estimates were calculated by simulating 1000 samples of the coefficients through Monte Carlo simulations, assuming a multivariate normal distribution. In a sensitivity analysis, we changed the number of degrees of freedom to model the non-linearity of temperature to 4 knots placed at the 10th, 50th, 75th and 90th percentiles. All analyses were conducted using R (version 4.2.1) and the *dlnm* package [31].

## Results

**Fig. 1:**
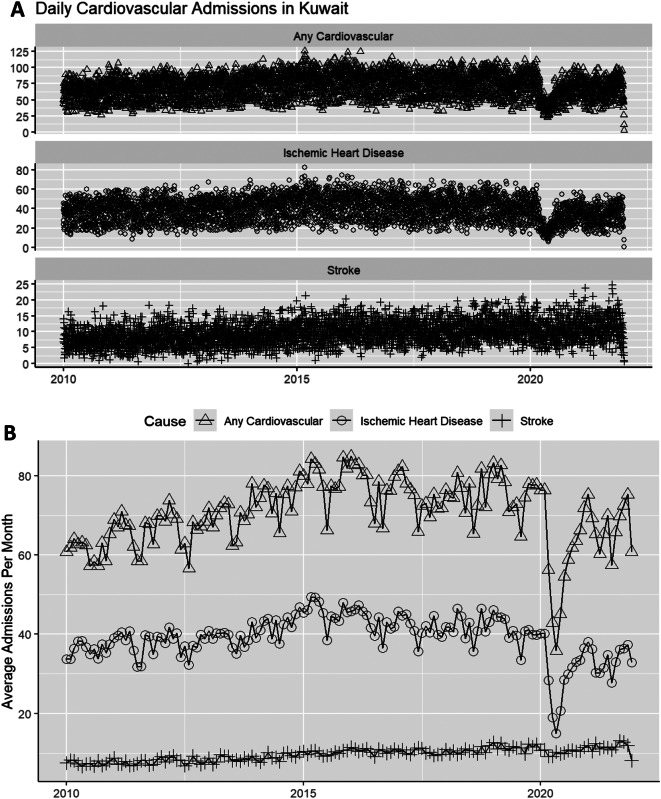
Daily and monthly average cardiovascular hospital admissions in Kuwait (2010–2021)

Daily hospitalizations significantly decreased in 2020 (Fig. 1) due to major disruptions in the healthcare system caused by COVID-19. Therefore, only hospitalization data from 2010 to 2019 were used for baseline analysis. During this period, a total of 263,182 cardiovascular disease cases were recorded, with 148,485 attributed to IHD and 34,240 to stroke. On average, there were 72.1 (± 17.5) cardiovascular admissions every day; 40.7 (± 11.6) for IHD and 9.4 (± 3.4) for stroke.

**Fig. 2:**
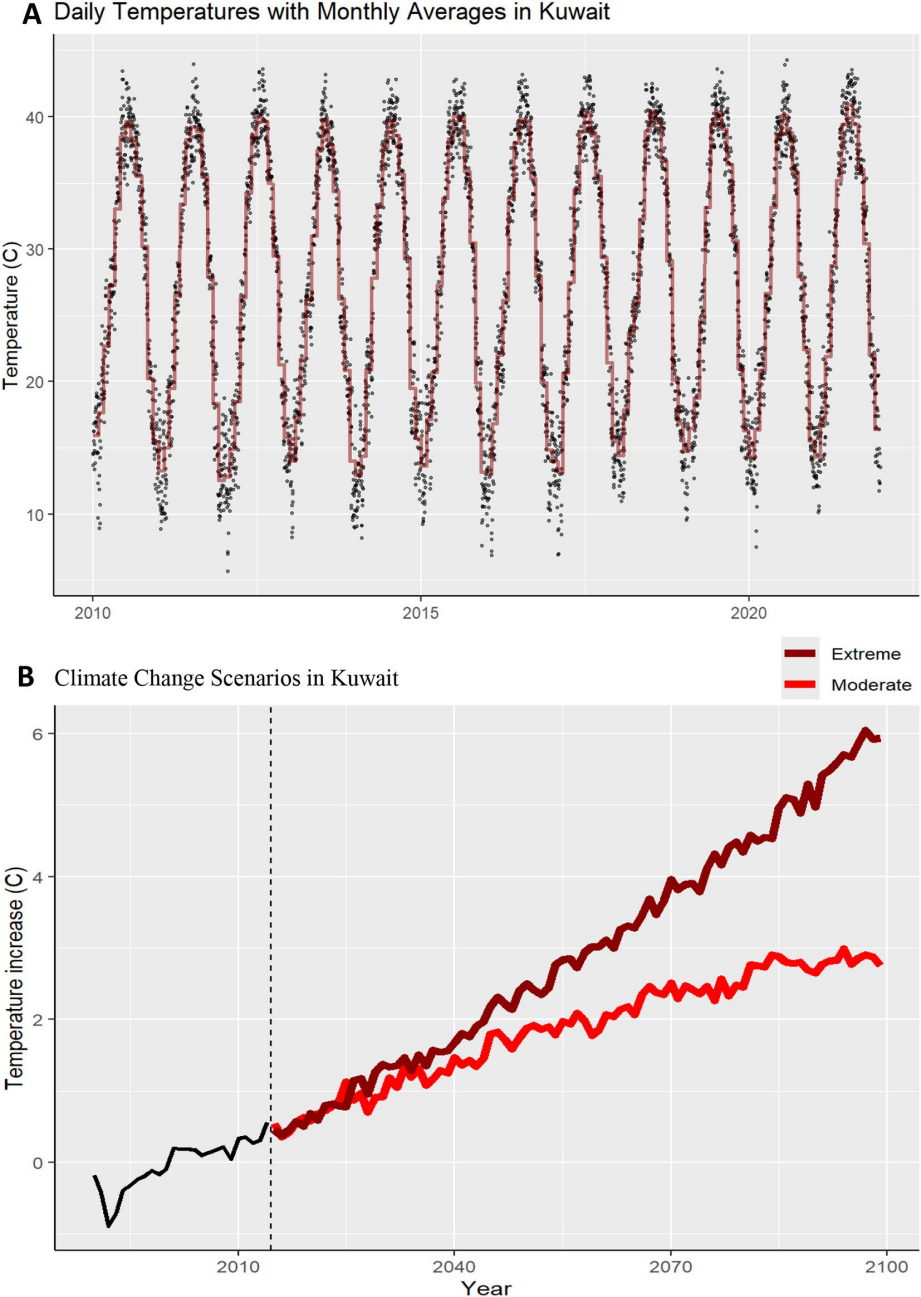
Daily temperature (2010-2021) and projected climate change scenarios (up to the year 2100) in Kuwait

Daily historical (2010–2019) temperature data in Kuwait followed a consistent seasonal trend with an overall upward trend (Fig. 2A). During this period, Kuwait had a mean average temperature of 27.4 °C, a minimum of 5.7 °C and a maximum of 44 °C. Future temperatures are projected to increase by 1.80 ± 0.58 °C by mid-century (2050–2059) and by 2.70 ± 0.73 °C by the end of the century (2090–2099) in the moderate SSP2-4.5 scenario, compared to the first decade of this millennium (2000–2009). In the extreme scenario, temperatures are expected to rise by 2.57 ± 0.67 °C by mid-century and by 5.54 ± 1.27 °C by late-century (Fig. 2B). In both scenarios, temperatures in Kuwait are expected to increase rapidly until mid-century, with the rate of warming slowing in the moderate scenario thereafter.

**Fig. 3:**
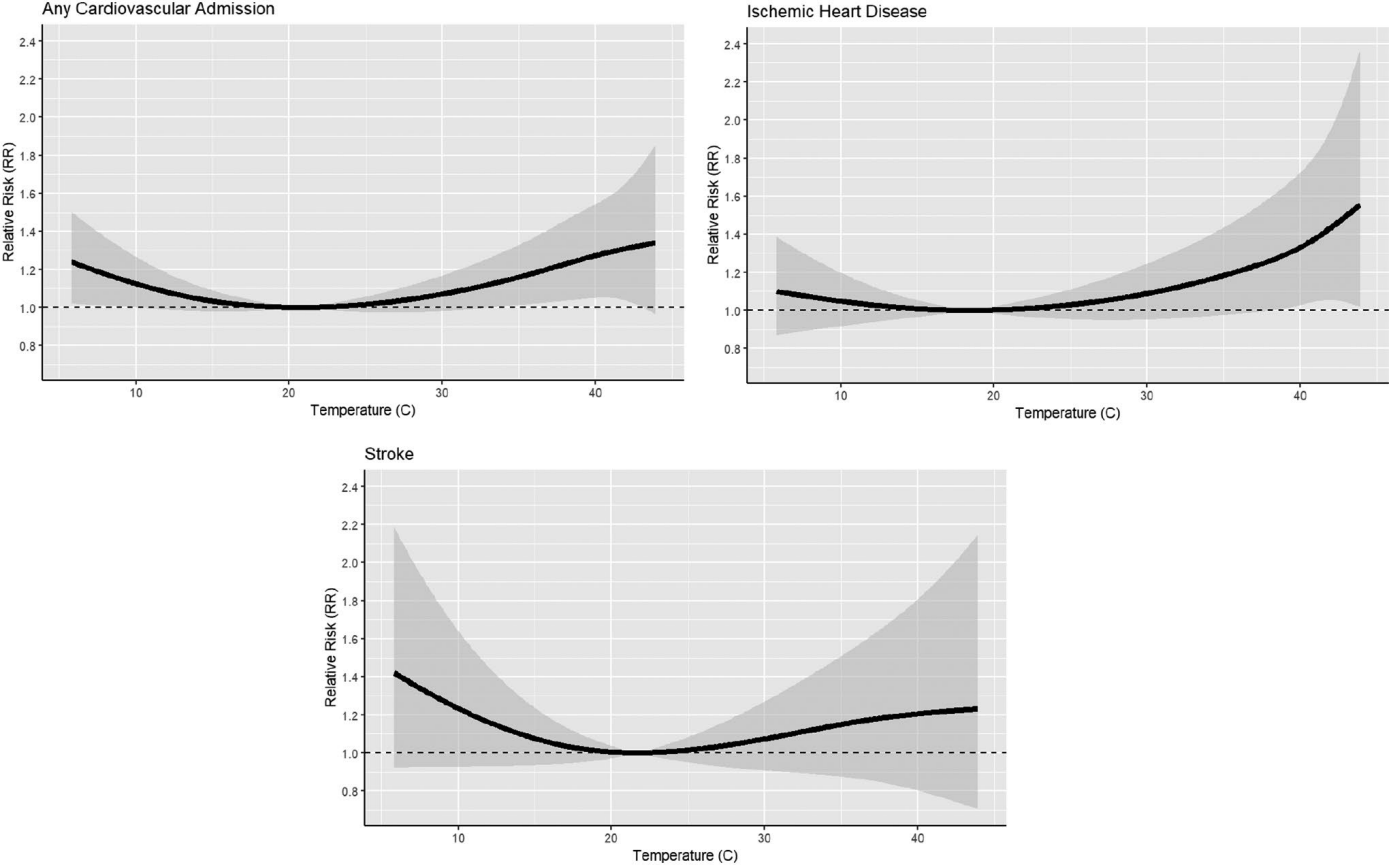
Exposure response relationship between temperature and any cardiovascular, ischemic heart disease and stroke hospitalizations in Kuwait (2010–2019)

Table 1 shows the relative risks of hospitalizations for any cardiovascular, IHD, and stroke at extreme temperature levels (41 °C, 42 °C, and 43 °C) compared to the MMTs (20.7 °C, 18.4 °C, and 21.7 °C for any cardiovascular event, IHD, and stroke, respectively) for the years 2010–2019. For any cardiovascular cause, the relative risk increased from 1.292 (95% CI: 1.051, 1.589) at 41 °C to 1.326 (95% CI: 1.006, 1.747) at 43 °C. A similar trend is observed for IHD, with relative risks increasing from 1.378 (95% CI: 1.046, 1.815) at 41 °C to 1.495 (95% CI: 1.042, 2.144) at 43 °C. For stroke, the relative risk ranged from 1.212 (95% CI: 0.780, 1.882) at 41 °C to 1.225 (95% CI: 0.730, 2.056) at 43 °C. Attributable admissions for all days in the 2010–2019 decade with temperatures above the MMT were 20,569 (95% eCI: 3,128, 35,757) admissions of any cardiovascular disease, 13,741 (95% eCI: −1,941, 26,169) admissions of IHD, and 2,379 (95% eCI: −2,533, 6,287) admissions of stroke. Increasing the degrees of freedom of temperature to 4 knots did not change the results meaningfully. Exposure response relationships are shown in Fig. 3.

**Table 1:** Relative risk for 41, 42 and 43 °C compared to minimum morbidity temperature (MMT) and heat attributable admissions for any Cardiovascular, Ischemic Heart Disease and Stroke (2010–2019)

	Any Cardiovascular	Ischemic Heart Disease	Stroke
*Relative risk*			
MMT	20.7 °C	18.4 °C	21.7 °C
41 °C vs. MMT	1.292 [1.051, 1.589]	1.378 [1.046, 1.815]	1.212 [0.780, 1.882]
42 °C vs. MMT	1.309 [1.036, 1.654]	1.433 [1.052, 1.951]	1.219 [0.756, 1.965]
43 °C vs. MMT	1.326 [1.006, 1.747]	1.495 [1.042, 2.144]	1.225 [0.730, 2.056]
*Attributable admissions for all hot days above the MMT*			
Attributable cases	20,569 [3128, 35757]	13,741 [-1941, 26169]	2379 [-2533, 6287]

**Fig. 4:**
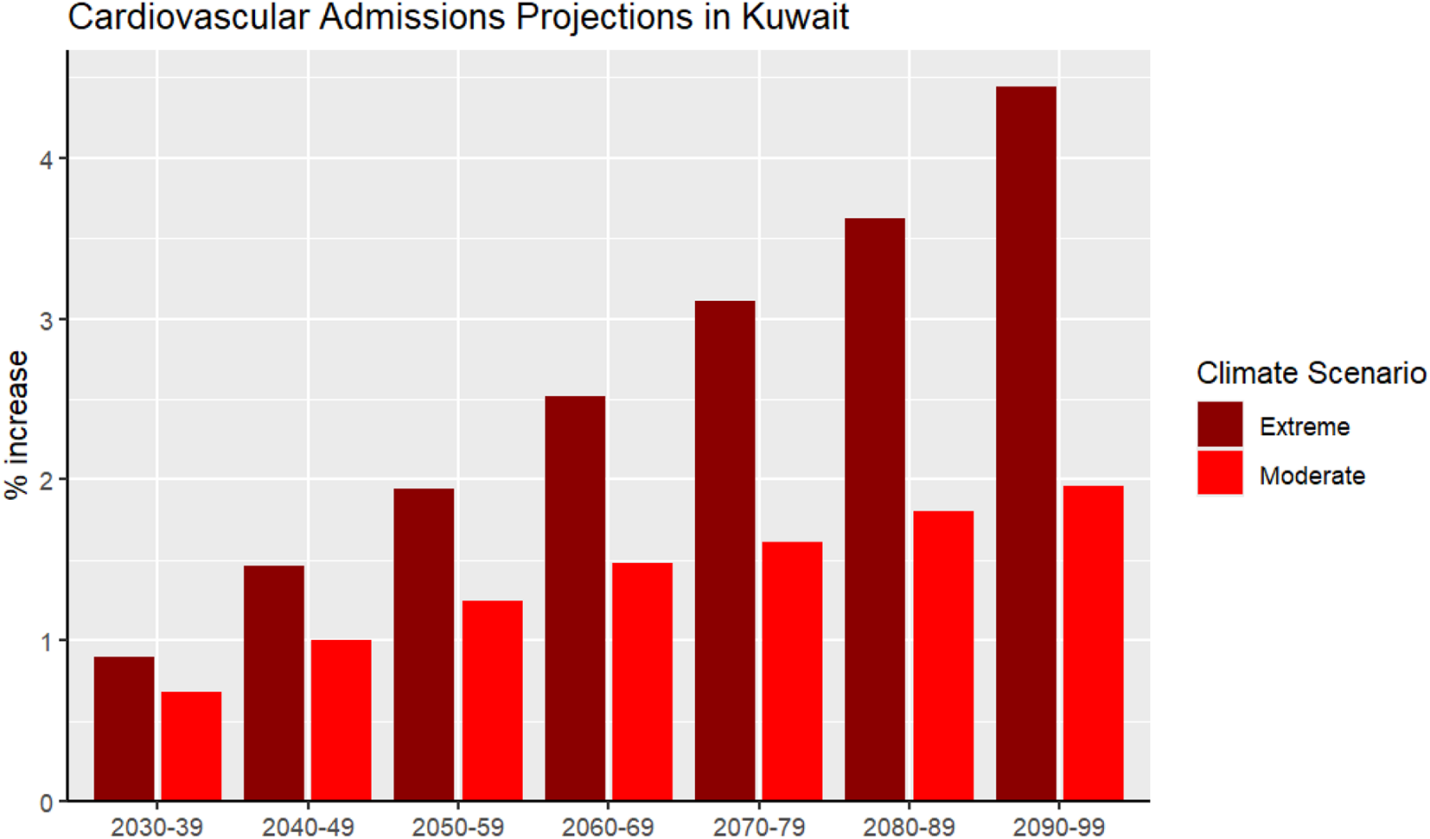
Projected percentage increase in cardiovascular hospital admissions under extreme and moderate climate scenarios in Kuwait up to the year 2100

Figure 4 presents the projected percentage increase in all cardiovascular admissions for multiple future time periods under two climate scenarios, moderate (SSP2-4.5) and extreme (SSP5-8.5) compared to the baseline decade of 2010–2019. Under the moderate scenario, cardiovascular admissions are expected to gradually increase over time, starting with a 0.68% increase (− 0.04, 1.54) for 2030–2039, reaching 1.96% (− 0.70, 4.74) by 2090–2099. In the more extreme scenario, the projected increases in cardiovascular admissions are higher compared to the moderate scenario. For 2030–2039, admissions are expected to increase by 0.89% (− 0.10, 2.04), rising to 4.44% (− 4.07, 11.28) by 2090–2099.

## Discussion

This study showed the impact of rising temperatures on cardiovascular health in Kuwait, historically and in the future. The baseline data from 2010 to 2019 showed an association between extreme heat and increased hospital admissions for cardiovascular diseases, ischemic heart disease, and stroke. Relative risks of hospitalization due to these conditions rose consistently with increasing temperatures above 40 °C. The trend of these temperatures is projected to worsen in future decades. Our projections indicate that, under both moderate and extreme climate change scenarios, the burden of cardiovascular disease in Kuwait will continue to rise, with substantial increases in hospital admissions expected by the end of the century. These conclusions lead to two immediate implications: (1) there is an urgent need for climate adaptation, and (2) the health system must prepare to mitigate the future health burden caused by extreme heat in Kuwait.

There are about 5 million deaths each year that could be attributed to non-optimal temperatures globally [32]. Extreme temperatures are now recognized as an emerging risk factor [33], and may be responsible for 1 in every 100 cardiovascular deaths [34]. Despite the fact that the connection between temperature and mortality has been extensively studied in the literature, comparatively fewer studies have been conducted on the effects on morbidity, particularly in the global south. In a systematic review, researchers found that short-term cold exposure in the general population increased the risk of cardiovascular admissions, but there was no significant effect from heat exposure [35]. Some studies from Europe showed no connection between heat exposure and cardiovascular hospitalizations [36–38], but other studies from Australia [39], Vietnam [40], and the US [41] reported a higher risk from extremely high temperatures. A systematic review of 14 studies analyzing the effects of temperature on acute myocardial infarction showed that in large and relatively well-controlled studies, both hot and cold weather increased the incidence of heart attacks [42]. The effects of temperature on health can vary greatly depending on geography, climate, demographics, and population acclimatization. Despite the rapid heating in the Middle East, there is paucity of research papers investigating the effects of temperatures on hospital admissions. A paper in Iran found significant increases of acute myocardial infarction admissions, especially during the warm season [43]. In Cyprus, a study reported an increase in all-cause hospital admissions [44] and recently for cardiovascular and respiratory causes [45]. In this local epidemiology study in Kuwait we found that heat is now associated with cardiovascular disease hospitalization and will likely worsen in the future.

Healthcare systems across the world are facing intense pressure as a result of aging populations with intricate healthcare requirements, expanding population size, migration, and treatments that are becoming increasingly costly [46, 47]. The COVID-19 pandemic has clearly shown the limited capability of many healthcare systems to absorb large disruptions once a threshold has been reached. The potential health burden of climate change is quickly becoming an area of interest around the world with emerging studies connecting heat with serious health consequences [23, 48, 49] including some of the recent research specific to Kuwait that examine historically observed effects [16] and future projections [15]. Currently, hospitals do not exactly know the potential impacts of climate change on bed capacity, staff planning, or resource allocation. Mid- and high-level hospital decision-makers will be facing increasingly difficult decisions to make evidence-based adaptation plans.

There are a number of proposed biological mechanisms in which heat can result in adverse cardiovascular outcomes. First, when the core body temperature increases, excessive sweating and peripheral vasodilation will lead to dehydration and electrolyte imbalances, and can trigger a sympathetic response, leading to tachycardia [50, 51]. Additionally, there is evidence of increased risk of ischemia or ruptured atherosclerotic plaque due to increased hypercoagulability when the temperatures are increasing [52]. Extreme heat is also implicated in cellular endothelial dysfunction and protein conformational changes, leading to systemic inflammation [53]. Put together, these physiological responses can add an additional strain to the heart and could eventually lead to fatal outcomes, especially among those with existing comorbidities [50, 54]. More mechanistic research is needed to carefully examine how these pathways interact under varying heat exposure conditions.

This study has a number of limitations. First, we only included 10 years of cardiovascular data from 2010 to 2019. We had to exclude the COVID years of 2020 and 2021 because they may not be representative of the actual burden of cardiovascular disease. Second, we assumed there will be no adaptation in the future. This is unlikely to hold. However, making assumptions on future technological, infrastructural and physical adaptations hold many uncertainties and is not a task one would want to undertake. Third, we did not account for population ageing. Recent studies showed that climate-mortality projections are more amplified when accounting for ageing [55, 56]. Fourth, we do not know what the future composition of the population might be. For example, we could see larger changes in migration. In this analysis, we did not account for such changes and assumed a static composition in the future. Finally, in estimating the baseline relationship between temperature and hospitalizations, we assumed that the ambient temperature averaged from a monitoring station serves as a proxy for personal exposure in microenvironments. However, this may not accurately reflect the actual exposure of interest.

## Conclusion

As the evidence mounts, tackling climate change is not only an environmental imperative but also a public health intervention, especially for hospital admissions. Addressing climate-induced risks on common causes like cardiovascular diseases will require robust health infrastructure capable of responding to increased demands, and integrated policies that address both health and environmental sustainability. By acting on the intricate links between climate change and cardiovascular health, hot places like Kuwait can better protect those most at risk from the increasingly projected harsh climates in a changing world.

## Data Availability

Hospitalization and Kuwait station temperature data required special approvals to obtain and can’t be publicly shared. future temperature model data is publicly available.

## References

[1] World Bank. Population, total - Middle East & North Africa. 2023.https://data.worldbank.org/indicator/SP.POP.TOTL?locations=ZQ (accessed 1 Dec2023).

[2] Lelieveld J, Hadjinicolaou P, Kostopoulou E, et al. Climate change and impacts in the Eastern Mediterranean and the Middle East. Clim Change. 2012;114:667–87. 10.1007/s10584-012-0418-4. 25834296 10.1007/s10584-012-0418-4 PMC4372776

[3] Pal JS, Eltahir EAB. Future temperature in southwest Asia projected to exceed a threshold for human adaptability. Nat Clim Chang. 2016;6:197–200. 10.1038/nclimate2833.

[4] Sharma A, Andhikaputra G, Wang Y-C. Heatwaves in south asia: characterization, consequences on human health, and adaptation strategies. Atmosphere. 2022;13:734. 10.3390/atmos13050734.

[5] Alwadi Y, Alahmad B. The burden of heat in arid regions of the Middle East: an analysis from Jordan and Kuwait. Environ Res: Health. 2024;2:035006. 10.1088/2752-5309/ad54e5.

[6] Domeisen DIV, Eltahir EAB, Fischer EM, et al. Prediction and projection of heatwaves. Nat Rev Earth Environ Published Online First: 13 Dec. 2022. 10.1038/s43017-022-00371-z.

[7] Intergovernmental Panel Climate Change. Climate Change 2021: the physical science basis - the Working Group I contribution to the Sixth Assessment Report. IPCC WG I; 2021.

[8] Lelieveld J, Proestos Y, Hadjinicolaou P, et al. Strongly increasing heat extremes in the Middle East and North Africa (MENA) in the 21st century. Clim Change. 2016;137:245–60. 10.1007/s10584-016-1665-6.

[9] Zittis G, Hadjinicolaou P, Klangidou M, et al. A multi-model, multi-scenario, and multi-domain analysis of regional climate projections for the Mediterranean. Reg Environ Change. 2019;19:2621–35. 10.1007/s10113-019-01565-w.

[10] Zittis G, Hadjinicolaou P, Fnais M, et al. Projected changes in heat wave characteristics in the eastern Mediterranean and the Middle East. Reg Environ Change. 2016;16:1863–76. 10.1007/s10113-014-0753-2.

[11] Ahmadalipour A, Moradkhani H, Kumar M. Mortality risk from heat stress expected to hit poorest nations the hardest. Clim Change. 2019;152:569–79. 10.1007/s10584-018-2348-2.

[12] Merlone A, Al-Dashti H, Faisal N, et al. Temperature extreme records: World Meteorological Organization metrological and meteorological evaluation of the 54.0°C observations in Mitribah, Kuwait and Turbat, Pakistan in 2016/2017. Int J Climatol Published Online First: 17 June. 2019. 10.1002/joc.6132.

[13] Alahmad B, Al-Hemoud A, Al-Bouwarthan M, et al. Extreme heat and work injuries in Kuwait’s hot summers. Occup Environ Med. 2023;80:347–52. 10.1136/oemed-2022-108697. 37068948 10.1136/oemed-2022-108697 PMC10314047

[14] Alahmad B, Shakarchi A, Alseaidan M, et al. The effects of temperature on short-term mortality risk in Kuwait: a time-series analysis. Environ Res. 2019;171:278–84. 10.1016/j.envres.2019.01.029. 30703623 10.1016/j.envres.2019.01.029

[15] Alahmad B, Vicedo-Cabrera AM, Chen K, et al. Climate change and health in Kuwait: temperature and mortality projections under different climatic scenarios. Environ Res Lett. 2022;17:074001. 10.1088/1748-9326/ac7601.

[16] Alahmad B, Shakarchi AF, Khraishah H, et al. Extreme temperatures and mortality in Kuwait: who is vulnerable? Sci Total Environ. 2020;732:139289. 10.1016/j.scitotenv.2020.139289. 32438154 10.1016/j.scitotenv.2020.139289

[17] Alahmad B, Ali H, Alwadi Y, et al. Combined impact of heat and dust on diabetes hospitalization in Kuwait. BMJ Open Diabetes Res Care. 2024;12. 10.1136/bmjdrc-2024-004320. 10.1136/bmjdrc-2024-004320 PMC11367401 39209775

[18] Alahmad B, Khraishah H, Shakarchi AF, et al. Cardiovascular mortality and exposure to heat in an inherently hot region: implications for climate change. Circulation. 2020;141:1271–3. 10.1161/CIRCULATIONAHA.119.044860. 32223316 10.1161/CIRCULATIONAHA.119.044860 PMC9060422

[19] World Health Organization. Kuwait [Country overview]. 2024 data.who.int. 2024.https://data.who.int/countries/414 (accessed 15 Nov2024).

[20] Alarouj M, Bennakhi A, Alnesef Y, et al. Diabetes and associated cardiovascular risk factors in the state of Kuwait: the first national survey. Int J Clin Pract. 2013;67:89–96. 10.1111/ijcp.12064. 23241053 10.1111/ijcp.12064

[21] Awad AI, Alsaleh FM. 10-year risk estimation for type 2 diabetes mellitus and coronary heart disease in Kuwait: a cross-sectional population-based study. PLoS ONE. 2015;10:e0116742. 10.1371/journal.pone.0116742. 25629920 10.1371/journal.pone.0116742 PMC4309592

[22] Alibrahim A, AlAjeel A. Noncommunicable diseases and hospital utilization in Kuwait: a generalizable approach using the world health survey. Med Princ Pract Published Online First: 25 August. 2022. 10.1159/000526673. 10.1159/000526673 PMC9801364 36007490

[23] Ebi KL, Capon A, Berry P, et al. Hot weather and heat extremes: health risks. Lancet. 2021;398:698–708. 10.1016/S0140-6736(21)01208-3. 34419205 10.1016/S0140-6736(21)01208-3

[24] O’Neill BC, Tebaldi C, van Vuuren DP, et al. The scenario model intercomparison project (scenariomip) for CMIP6. Geosci Model Dev. 2016;9:3461–82. 10.5194/gmd-9-3461-2016.

[25] Eyring V, Bony S, Meehl GA, et al. Overview of the coupled model Intercomparison Project Phase 6 (CMIP6) experimental design and organization. Geosci Model Dev. 2016;9:1937–58. 10.5194/gmd-9-1937-2016.

[26] Lange S. Trend-preserving bias adjustment and statistical downscaling with ISIMIP3BASD (v1.0). Geosci Model Dev. 2019;12:3055–70. 10.5194/gmd-12-3055-2019.

[27] Gasparrini A, Guo Y, Hashizume M, et al. Mortality risk attributable to high and low ambient temperature: a multicountry observational study. Lancet. 2015;386:369–75. 10.1016/S0140-6736(14)62114-0. 26003380 10.1016/S0140-6736(14)62114-0 PMC4521077

[28] Gasparrini A, Guo Y, Sera F, et al. Projections of temperature-related excess mortality under climate change scenarios. Lancet Planet Health. 2017;1:e360–7. 10.1016/S2542-5196(17)30156-0. 29276803 10.1016/S2542-5196(17)30156-0 PMC5729020

[29] Alahmad B, Yuan Q, Achilleos S, et al. Evaluating the temperature-mortality relationship over 16 years in Cyprus. J Air Waste Manag Assoc. 2024;1–10. 10.1080/10962247.2024.2345637. 10.1080/10962247.2024.2345637 38718302

[30] Vicedo-Cabrera AM, Sera F, Gasparrini A. Hands-on Tutorial on a modeling Framework for projections of Climate Change impacts on Health. Epidemiology. 2019;30:321–9. 10.1097/EDE.0000000000000982. 30829832 10.1097/EDE.0000000000000982 PMC6533172

[31] Gasparrini A. Distributed lag Linear and Non-linear models in R: the Package dlnm. J Stat Softw. 2011;43:1–20. 22003319 PMC3191524

[32] Zhao Q, Guo Y, Ye T, et al. Global, regional, and national burden of mortality associated with non-optimal ambient temperatures from 2000 to 2019: a three-stage modelling study. Lancet Planet Health. 2021;5:e415–25. 10.1016/S2542-5196(21)00081-4. 34245712 10.1016/S2542-5196(21)00081-4

[33] GBD 2019 Risk Factors Collaborators. Global burden of 87 risk factors in 204 countries and territories, 1990–2019: a systematic analysis for the global burden of Disease Study 2019. Lancet. 2020;396:1223–49. 10.1016/S0140-6736(20)30752-2. 33069327 10.1016/S0140-6736(20)30752-2 PMC7566194

[34] Alahmad B, Khraishah H, Royé D, et al. Associations between Extreme temperatures and Cardiovascular cause-specific mortality: results from 27 countries. Circulation. 2023;147:35–46. 10.1161/CIRCULATIONAHA.122.061832. 36503273 10.1161/CIRCULATIONAHA.122.061832 PMC9794133

[35] Phung D, Thai PK, Guo Y, et al. Ambient temperature and risk of cardiovascular hospitalization: an updated systematic review and meta-analysis. Sci Total Environ. 2016;550:1084–102. 10.1016/j.scitotenv.2016.01.154. 26871555 10.1016/j.scitotenv.2016.01.154

[36] Iñiguez C, Royé D, Tobías A. Contrasting patterns of temperature related mortality and hospitalization by cardiovascular and respiratory diseases in 52 Spanish cities. Environ Res. 2021;192:110191. 10.1016/j.envres.2020.110191. 32980302 10.1016/j.envres.2020.110191

[37] Michelozzi P, Accetta G, De Sario M, et al. High temperature and hospitalizations for cardiovascular and respiratory causes in 12 European cities. Am J Respir Crit Care Med. 2009;179:383–9. 10.1164/rccm.200802-217OC. 19060232 10.1164/rccm.200802-217OC

[38] Ponjoan A, Blanch J, Alves-Cabratosa L, et al. Effects of extreme temperatures on cardiovascular emergency hospitalizations in a Mediterranean region: a self-controlled case series study. Environ Health. 2017;16:32. 10.1186/s12940-017-0238-0. 28376798 10.1186/s12940-017-0238-0 PMC5379535

[39] Nitschke M, Tucker GR, Hansen AL, et al. Impact of two recent extreme heat episodes on morbidity and mortality in Adelaide, South Australia: a case-series analysis. Environ Health. 2011;10:42. 10.1186/1476-069X-10-42. 21592410 10.1186/1476-069X-10-42 PMC3116460

[40] Thu Dang TA, Wraith D, Bambrick H, et al. Short - term effects of temperature on hospital admissions for acute myocardial infarction: a comparison between two neighboring climate zones in Vietnam. Environ Res. 2019;175:167–77. 10.1016/j.envres.2019.04.023. 31128426 10.1016/j.envres.2019.04.023

[41] Lin S, Luo M, Walker RJ, et al. Extreme high temperatures and hospital admissions for respiratory and cardiovascular diseases. Epidemiology. 2009;20:738–46. 10.1097/EDE.0b013e3181ad5522. 19593155 10.1097/EDE.0b013e3181ad5522

[42] Bhaskaran K, Hajat S, Haines A, et al. Effects of ambient temperature on the incidence of myocardial infarction. Heart. 2009;95:1760–9. 10.1136/hrt.2009.175000. 19635724 10.1136/hrt.2009.175000

[43] Mohammadi R, Khodakarim S, Alipour A, et al. Association between Air Temperature and Acute Myocardial Infarction hospitalizations in Tehran, Iran: a time-stratified case-crossover. Int J Occup Environ Med. 2017;8:143–52. 10.15171/ijoem.2017.1069. 28689211 10.15171/ijoem.2017.1069 PMC6679627

[44] Pantavou K, Giallouros G, Philippopoulos K, et al. Thermal conditions and hospital admissions: analysis of Longitudinal Data from Cyprus (2009–2018). Int J Environ Res Public Health. 2021;18. 10.3390/ijerph182413361. 10.3390/ijerph182413361 PMC8702178 34948967

[45] Wang Y, Achilleos S, Salameh P, et al. Temperature and hospital admissions in the Eastern Mediterranean: a case study in Cyprus. Environ Res: Health. 2024;2:025004. 10.1088/2752-5309/ad2780.

[46] Song Z, Ferris TG. Baby boomers and beds: a demographic challenge for the ages. J Gen Intern Med. 2018;33:367–9. 10.1007/s11606-017-4257-x. 29273896 10.1007/s11606-017-4257-x PMC5834972

[47] Bone AE, Gomes B, Etkind SN, et al. What is the impact of population ageing on the future provision of end-of-life care? Population-based projections of place of death. Palliat Med. 2018;32:329–36. 10.1177/0269216317734435. 29017018 10.1177/0269216317734435 PMC5788077

[48] Aström C, Orru H, Rocklöv J, et al. Heat-related respiratory hospital admissions in Europe in a changing climate: a health impact assessment. BMJ Open. 2013;3. 10.1136/bmjopen-2012-001842. 10.1136/bmjopen-2012-001842 PMC3563142 23355662

[49] Watson KE, Gardiner KM, Singleton JA. The impact of extreme heat events on hospital admissions to the Royal Hobart Hospital. J Public Health. 2020;42:333–9. 10.1093/pubmed/fdz033. 10.1093/pubmed/fdz033 31220305

[50] Khraishah H, Alahmad B, Ostergard RL, et al. Climate change and cardiovascular disease: implications for global health. Nat Rev Cardiol. 2022;19:798–812. 10.1038/s41569-022-00720-x. 35672485 10.1038/s41569-022-00720-x

[51] Epstein Y, Yanovich R, Heatstroke. N Engl J Med. 2019;380:2449–59. 10.1056/NEJMra1810762. 31216400 10.1056/NEJMra1810762

[52] Peters A, Schneider A. Cardiovascular risks of climate change. Nat Rev Cardiol. 2021;18:1–2. 10.1038/s41569-020-00473-5. 33169005 10.1038/s41569-020-00473-5 PMC7649889

[53] Zheng S, Zhu W, Shi Q, et al. Effects of cold and hot temperature on metabolic indicators in adults from a prospective cohort study. Sci Total Environ. 2021;772:145046. 10.1016/j.scitotenv.2021.145046. 33581536 10.1016/j.scitotenv.2021.145046

[54] Desai Y, Khraishah H, Alahmad B. Heat and the heart. Yale J Biol Med. 2023;96:197–203. 10.59249/HGAL4894. 37396980 10.59249/HGAL4894 PMC10303253

[55] Chen K, Vicedo-Cabrera AM, Dubrow R. Projections of ambient temperature- and Air Pollution-related mortality Burden under Combined Climate Change and Population aging scenarios: a review. Curr Environ Health Rep. 2020;7:243–55. 10.1007/s40572-020-00281-6. 32542573 10.1007/s40572-020-00281-6

[56] Chen K, de Schrijver E, Sivaraj S, et al. Impact of population aging on future temperature-related mortality at different global warming levels. Nat Commun. 2024;15:1796. 10.1038/s41467-024-45901-z. 38413648 10.1038/s41467-024-45901-z PMC10899213

